# Deep Structural Analysis of Myriads of Omicron Sub-Variants Revealed Hotspot for Vaccine Escape Immunity

**DOI:** 10.3390/vaccines11030668

**Published:** 2023-03-15

**Authors:** Valeria Gerardi, Mohammed A. Rohaim, Rania F. El Naggar, Mustafa O. Atasoy, Muhammad Munir

**Affiliations:** 1 Division of Biomedical and Life Sciences, Faculty of Health and Medicine, Lancaster University, Lancaster LA1 4YG, UK; 2 Department of Virology, Faculty of Veterinary Medicine, Cairo University, Giza 12211, Egypt; 3 Department of Virology, Faculty of Veterinary Medicine, University of Sadat City, Sadat 32897, Egypt

**Keywords:** Omicron variant, hACE2 receptor, RBD mutations, structural mapping, sub-variants, vaccine

## Abstract

The emergence of the Omicron variant has reinforced the importance of continued SARS-CoV-2 evolution and its possible impact on vaccine effectiveness. Specifically, mutations in the receptor-binding domain (RBD) are critical to comprehend the flexibility and dynamicity of the viral interaction with the human agniotensin-converting enzyme 2 (hACE2) receptor. To this end, we have applied a string of deep structural and genetic analysis tools to map the substitution patterns in the S protein of major Omicron sub-variants (n = 51) with a primary focus on the RBD mutations. This head-to-head comparison of Omicron sub-variants revealed multiple simultaneous mutations that are attributed to antibody escape, and increased affinity and binding to hACE2. Our deep mapping of the substitution matrix indicated a high level of diversity at the N-terminal and RBD domains compared with other regions of the S protein, highlighting the importance of these two domains in a matched vaccination approach. Structural mapping identified highly variable mutations in the up confirmation of the S protein and at sites that critically define the function of the S protein in the virus pathobiology. These substitutional trends offer support in tracking mutations along the evolutionary trajectories of SAR-CoV-2. Collectively, the findings highlight critical areas of mutations across the major Omicron sub-variants and propose several hotspots in the S proteins of SARS-CoV-2 sub-variants to train the future design and development of COVID-19 vaccines.

## 1. Introduction

The coronavirus disease 2019 (COVID-19) emerged in Wuhan, China, and spread rapidly around the world guiding the World Health Organisation (WHO) to declare a Public Health Emergency [1]. The severe acute respiratory syndrome coronavirus 2 (SARS-CoV-2) belongs to the genus β-coronavirus, within the subfamily, *Orthocoronavirinae*, in the family *Coronaviridae*, order *Nidovirales*, and realm *Riboviria*. The SARS-CoV-2 virions are spherical in shape and 50–200 nm in diameter. The SARS-CoV-2 is a pathogenic enveloped virus with a linear positive-sense single-stranded RNA (+ssRNA; class 4 of Baltimore) of ~29.9 kB in size [2]. The genome encodes for nine open reading frames (ORFs) that translate to at least 27 proteins. The 5′ UTR, replication complex (ORF1a and ORF1b), spike (S), envelope (E), membrane (M), and nucleocapsid (N) genes, and 3′ UTR, nine non-structural ORFs, and poly(A)-tail make up its structural components. The polyprotein precursor pp1a (10 nsps) is encoded by the ORF1a gene, found in the 5′UTR. Pp1ab (16 nsps) is encoded by the ORF1b gene [2]. The total coding potential of the SARS-CoV-2 genome is ~7096 residues [3,4].

To initiate an infection, the S protein first interacts with the human angiotensin-converting 2 receptor (hACE2) and facilitates the viral entrance into the host cell [5,6,7]. The S protein is the largest protein, with 1273 amino acid residues in prototype SARS-CoV-2 Wuhan-Hu-1, and it splits into three subunits: the N-terminus signal peptide, S1 and S2. The receptor-binding domain (RBD) of the S protein binds to the host ACE2 receptor and has the ability to boost infectivity and facilitate escape from the vaccine-induced neutralising antibodies [8,9,10]. Therefore, major research interest has been focused on mutations found in the RBD. Some of these mutations, which are known to cause enhanced transmissibility, higher viral binding affinity, and antibody escape have also been reported in previous variants [11,12]. Because the S protein mediates the virus-hACE2 interaction, several mutations have accumulated in this protein due to ongoing immunological pressure [13].

The evolution of SARS-CoV-2 has been remarkable in its speed and complexity. The virus genome has undergone several mutations to adapt in the host system [5]. The rapid evolution of SARS-CoV-2 caused the emergence of new variants. The *WHO* has identified variants of concern (VOCs) and variants of interest (VOIs) throughout the evolutionary pattern of SARS-CoV-2. Now, the only VOC is Omicron. De-escalated variants include Alpha, Gamma, and Delta [14]. The first VOC was discovered in South Africa and Botswana; the Omicron variant (B.1.1.529) of SARS-CoV-2 was reported as a new variant by the *WHO* on 24 November 2021 and was categorized as a VOC on 26 November 2021 [15,16].

The BA.1 is the parental Omicron variant, and it presents a deficit of three amino acids compared with the Wuhan-Hu-1, with a total of 1270 amino acid residues [17]. The BA.1 carried N679K and P681H mutations near the four-residue insert (PRRA) at the boundary between the S1 and S2 subunits that facilitates the cleavage in the S protein and enhance fusion and virus infection. Like BA.1, the BA.2 presents a total of 1270 amino acids while BA.3 presents a deficit of six amino acids (1267aa) compared with the Wuhan-Hu-1, and most of the mutations are shared between BA.1 and BA.2, including 15 mutations within the RBD [18]. The Omicron sub-variants BA.4 and BA.5 were identified for the first time in South Africa in January and February 2022, respectively. The S proteins of both BA.4 and BA.5 are identical and include additional mutations 69-70del, L452R, F486V, and a wild-type amino acid at position Q493 compared with BA.2. BA.4 also exhibits L11F and P151S mutations in the Orf7b, and a triple amino acid deletion in the NSP1 (141-14Ddel). Compared with BA.4, the BA.5 has a M:D3N mutation and extra reversions at Orf6:D61 at nucleotide positions 26,858 and 27,259. The L452R mutation in BA.4 and BA5 has been linked to a high receptor binding affinity, resulting in increased infectivity [19]. The resemblance of these lineages suggests a possible relationship during a recombination event.

Current antiviral drugs and COVID-19 vaccines may no longer be as effective against Omicron, particularly the new sub-variants [20]. The emergence of the Omicron variant has been explained in three ways: silent evolution in a population with limited sequencing; long-term evolution in one or a few people with chronic infection; or evolution in other species, particularly rodents [21]. Due to its infectious and vaccine-escape alterations, the Omicron variant has created widespread anxiety and alarm around the globe. Most of the Omicron-specific mutations have unknown consequences, and their roles in viral transmission and escape of immunity remain to be determined. Additionally, persons who have already contracted another SARS-CoV-2 variant are susceptible to re-infection with the Omicron variant and can evade immunity induced by the previous infection [22]. 

There is an urgent need for in-depth research and a thorough knowledge of Omicron because it poses a major risk to public health and may jeopardize attempts to contain the COVID-19 pandemic. There is a continuous evolution for the Omicron sub-variants, leading to immune evasion, a high risk of reinfection, and high transmissibility [22]. The purpose of this research is to use a breadth of bioinformatics tools to analyse the substitution patterns in the S protein of major Omicron sub-variants, with particular attention to the RBD, in comparison with SARS-CoV-2 Wuhan-Hu-1. This study offers the first head-to-head comparison of the current Omicron sub-variants that would be useful in predicting future trajectories and studying the critical mutations of current and future Omicron sub-variants.

## 2. Materials and Methods

### 2.1. Complete Genome Sequence Acquisition

The genome sequences of SARS-CoV-2 Omicron variant sub-lineages were obtained in FASTA format from the Global Initiative on Sharing Avian Influenza Data (GISAID) EpiCoV database (https://gisaid.org/ accessed on 14 February 2023). A search option integrated within the GISAID was performed to identify Omicron sub-variants, and the nucleotide sequence was chosen and analysed. Only complete nucleotide coverage without un-sequenced nucleotides were selected for analysis. Information on selected variants, their strain name, accession ID, and country of sequence are shown in Table S1.

### 2.2. Extraction of the S Gene Sequences

The full-length S gene sequences of each variant was imported via SnapGene Viewer 6.1.2 in FASTA format. The nucleotide sequences were translated into amino acid sequences and the amino acid sequences of each variant were put into a notepad file and saved in FASTA format.

### 2.3. Amino Acid Analyses of the S Protein

The amino acid sequences of the S protein including the RBD were imported to BioEdit Sequence Alignment Editor; sequences were aligned and compared with Wuhan-Hu-1 as shown in Figure S1. Additionally, the key amino residues interacting with the hACE2 upon binding of the virus with the host cell were analysed. The tables were then extracted into two separate FASTA files to compare the mutations amongst variants and to construct WebLogo representations via WebLogo Online Tool (weblogo.berkeley.edu/logo.cgi, accessed on 14 February 2023).

### 2.4. RBD 3D Modelling for Amino Acid Substitutions

A cryo-electron microscopy 3D structure model of the S protein with RBD of the Wuhan-Hu-1 in the prefusion conformation was downloaded from RCSB Protein Data Bank (accession ID: 6VSB) [23,24]. Each variant was designated a colour and the substitutions were labeled using PyMOL software. Using PyMOL, the 3D structure model of the same protein was labelled according to the most conserved and the most variable residues in the analysed Omicron sub-variants.

### 2.5. Phylogenetic Analysis

The aligned FASTA sequences of the S protein and complete genome were imported into Molecular Evolutionary Genetics Analysis (MEGA11). Phylogenetic trees of the Omicron SARS-CoV-2 sub-variants based on the RBD, full length S protein, and complete genome were constructed using the using general time-reversible (GTR) model, which was selected using jModelTest [25], and trees were constructed using RaxML version 8.2.11 [26] with 1000 bootstrap replicates.

## 3. Results

### 3.1. Amino Acid Substitution Analyses

**Figure 1:**
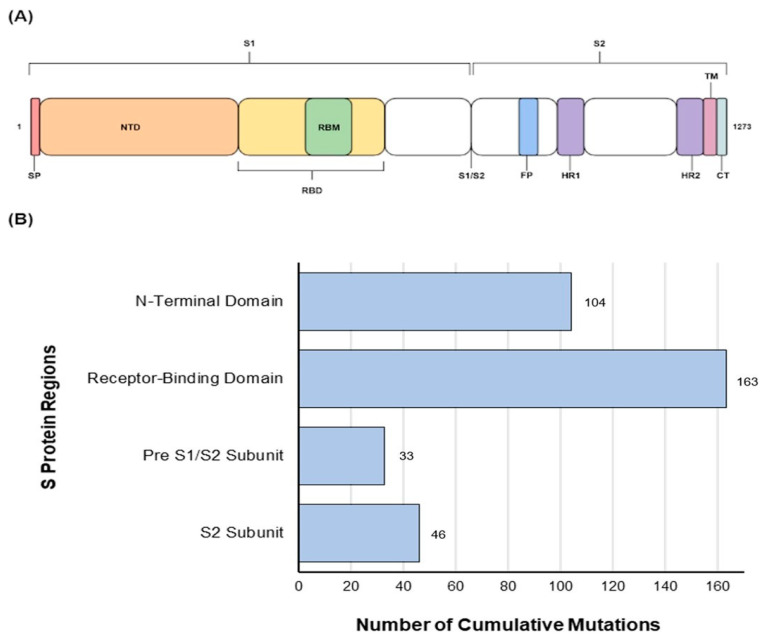
(**A**) Schematic presentation of different domains of the S protein. (**B**) Amino acid mutations (blue) of the residues of interest in the S gene of Omicron sub-variants studied compared with the Wuhan-Hu-1 variant.

Our results showed multiple amino acid substitutions within the S protein of Omicron sub-variants (Figure 1A). While substitutions were observed across the S protein, a higher level of mutations were identified within the RBD (n = 163) followed by the N-terminus domain of the S protein (n = 104) (1B) of all studied Omicron sub-variants (n = 51). These mutations at two immunologically important sites indicate continued evolution and validate the importance of the N-terminus and the RBD of S protein in future vaccine design.

**Figure 2:**
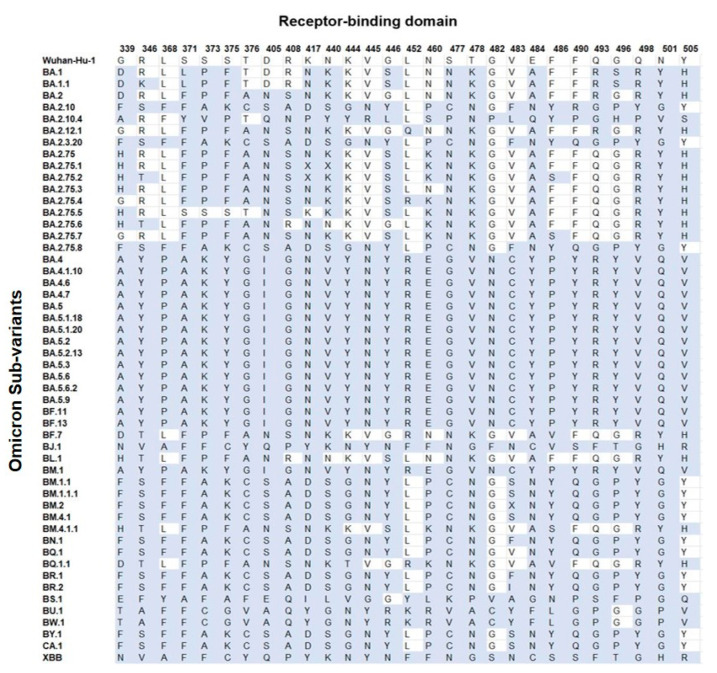
Amino acid mutations (blue) of the residues of interest in the RBD of each Omicron sub-variant compared with the Wuhan-Hu-1.

In addition, more mutations were seen in the N-terminal side of the protein, compared with the C-terminal domain while the pre S1/S2 domain was the most conserved domain of the S protein, in terms of cumulative substitutions (Figure 2). This region carried an average of 74.3% (circa 19/26 sites) of mutations per each Omicron sub-variant. Groups 4 and 5 and variants (BS.1, BA.2.10.4, BA.5.1.18) showed 100% (26/26) mutations, whereas BA.1 and BF.7 sub-variants carried only 7.7% (2/26) mutations compared with Wuhan-Hu-1.

**Figure 3:**
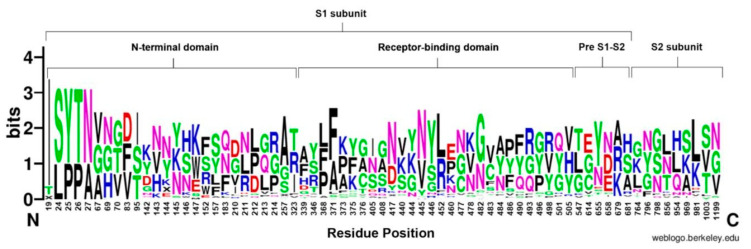
WebLogo representation of the amino acid substitutions within the S protein of SARS-CoV-2. Different domains of the S protein are labelled at the top of sequence conservation. The size of the letter (bits; Y axis) indicates the frequency of the amino acid substitutions at a certain residue position (X axis). Different residues at the same position are scaled according to their frequency and colored based on their amino acid characteristics.

Based on the mutational analysis in the RBD, it was plausible to categorize all Omicron sub-variants into groups (Figure 3 and Table S2). The BM.2 (group 3) and BR.2 differed from group 2 at position V483 and showed mutations at residues V483X/F/I, instead of V483S. The BA.2.75.1 differed from group 5 at positions K417N and N440K, showing mutations at residues K417X and N440X. The BA.2.75.2 differed from group 5 only at position K417N, showing substitution K417X. In addition, BQ.1 differed from group 6 only in position V483, showing no mutation, compared with group 6′s mutation at residue V483F; however, the XBB and BJ.1 differed only in position F486, showing F486S and F486V, respectively. The RBD region carried an average of 82% (circa 23/28 sites) of mutations per sub-variant. Groups 1 and 7 showed the highest number of mutations (100%; 28/28), whereas the lowest mutations were seen in BA.2.75.5 with 42.9% (12/28) mutations. This region carried an average of 74.3% (circa 19/26 sites) of mutations per sub-variant. 

### 3.2. Pre S1/S2 Subunit Substitutions 

Each sub-variant carrying the same mutations in the pre S1/S2 subunit have been grouped as shown in Table S4. BA.1 and BA.1.1 differed only at position H655, showing H655Y and H655T, respectively. BA.2 differed from group 3 only at position 655, having H655T instead of H655Y. The only two unique sub-variants were BS.1 and BA.2.10.4. This region carried an average of 89.2% (circa 5/6 sites) mutations per each sub-variant. Groups 1, 2, and 4 and sub-variants BS.1 and BA.2.10.4 showed 100% (6/6) mutations. The lowest similarity was seen in BA.2, with a rate of 50% (3/6).

### 3.3. S2 Subunit Substitutions

The sub-variants with the same mutations at the S2 subunit have been grouped as shown in Table S5. The sub-variant BA.4 differed from group 1 at position 764, having N764L instead of N764G. In addition, groups 3 and 4 and sub-variants BA.2.75 and BA.2.12.1 varied at position 764, showing N764K, N764L, and N764E, respectively. On the other hand, BA.2, BS.1 and BA.2.10.4 sub-variants showed unique S2 amino acids of interest. The S2 region carried an average of 76% (circa 7/9 sites) of mutations per sub-variant. Groups 1 and 5 and sub-variants BA.4 showed 100% mutations, while the lowest value was seen in group 4 and BA.2 with 33.3% (3/9) similarity.

### 3.4. Amino Acids Conservation and Variation

#### 3.4.1. N-Terminal Domain

The NTD showed a high number of conserved residues; in particular, position 19 was the most conserved site throughout the S proteins (90.2%) of all the studied Omicron sub-variants (n = 51). In addition, residues at 24, 25, 26, and 27 positions showed high conservation (68.6%). Additionally, substitutions were monitored at residues 142 and 210 and demonstrated the highest variability in the NTD (Figure 3).

#### 3.4.2. Receptor-Binding Domain

**Figure 4:**
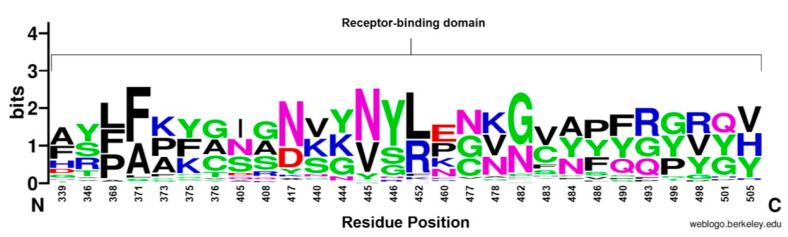
WebLogo representation of amino acid substitutions within the RBD of SARS-CoV-2. The size of the letter (bits; Y axis) indicates the frequency of the amino acid substitution at a certain residue position (X axis). Different residues at the same position are scaled according to their frequency. and colored based on their amino acid characteristics.

**Figure 5:**
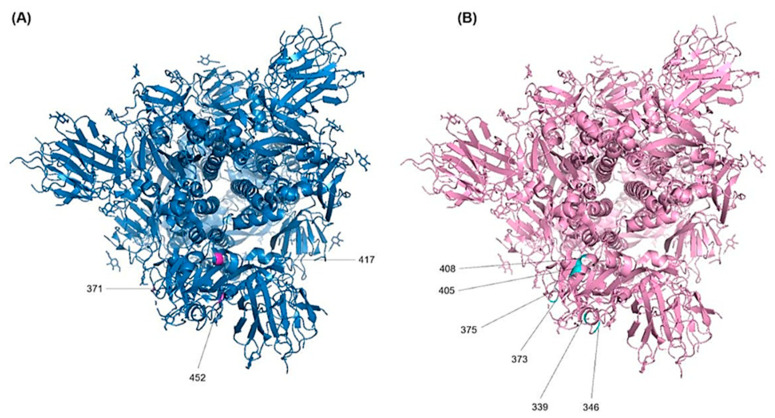
Localization of the RBD amino acid substitutions. The 3D structure- model of the S protein of the Wuhan-Hu-1 in the prefusion conformation (PDB ID: 6SVB) was used to annotate the most important resides. (**A**) Top view of the most conserved residues and (**B**) the most variable residues.

The RBD sequence showed the highest variability (Figure 4) across the length of the S protein. The most conserved residues were found at position 371 (58.8%), 417 (56.9%), 445 (56.9%), 446 (56.9%), 452 (51%), and 482 (56.9%) (Figure 5A). However, all other sites showed similar variabilities. The most variable sites were found at positions 339, 346, 373, 375, 405, and 408 (Figure 5B), where site 375 is the most variable site along the length of the S protein.

#### 3.4.3. Pre S1/S2 Subunit and S2 Subunit

The pre S1/S2 subunit showed the lowest number of cumulative substitutions, and the highest conservation (Figure 3). Overall, the pre S1/S2 subunit showed conservation particularly at site 655 (58.8%). Meanwhile, all variants showed substitution at position 679, Ala (33.3%), Arg (33.3%), and Lys (31.4%). However, only one sub-variant (BS.1) showed a unique Asn-to-Ser mutation at position 679. For the S2 subunit, the most conserved site was at site 981 with 52.3% out of the sub-variants. On the other hand, positions 764 and 856 showed high variability (Figure 3).

#### 3.4.4. D Visualization for the RBD Mutations

The localization of the substitutions within the RBD of the S protein (top view) were represented as shown in Figures S2–S7. Our results showed that all the sites analysed could be seen from the top view of the S protein using “the up conformation”, due to its position within the S protein. This highlights all residues, which were exposed on the RBD-hACE2 binding interface. 

### 3.5. Phylogeny and Evolution

**Figure 6:**
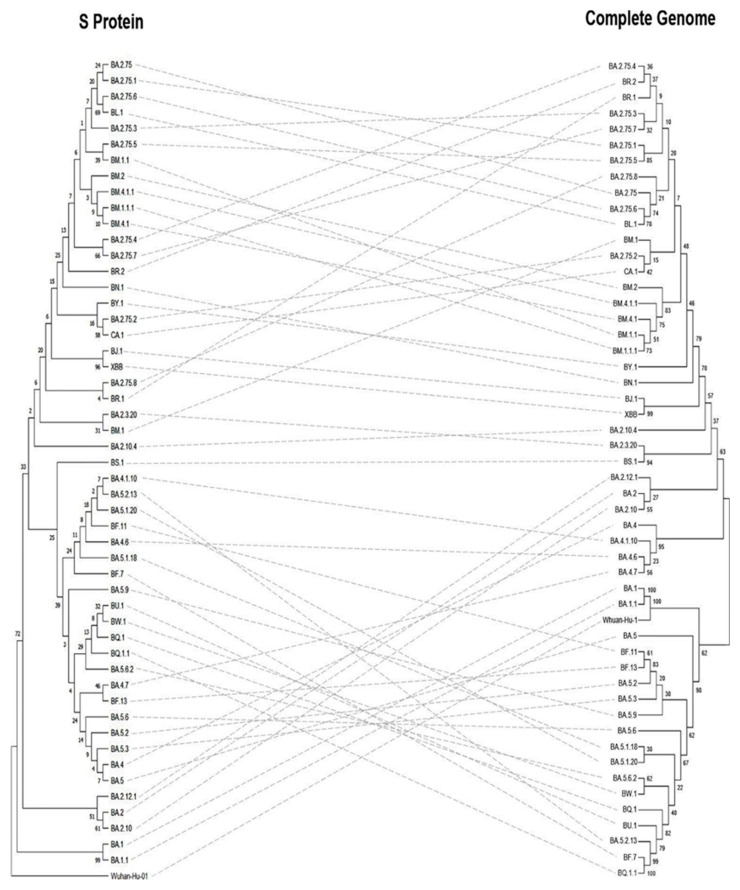
Phylogenetic tree of S protein (**left**) against the complete genome (**right**) of SARS-CoV-2 Omicron sub-variants. Sequences were obtained from the GISAID EpiCoV database as shown in Table S1.

The Neighbour-joining (NJ) tree for the complete genome, S protein, and RBD of the Omicron sub-variants revealed differing results. The phylogenetic analyses based on the S protein (Figure 6 left), the Wuhan-Hu-1 was the root for the tree, the common ancestor. The BA.1 and BA.1.1 evolved directly from Wuhan-Hu-1 showed 99% confidence and clustered together, whereas the complete genome analysis showed 100% evidence. The BJ.1 and XBB sub-variants showed a 96% confidence value based on the S protein analysis, while it was 99% with the complete genome. Meanwhile, BA.2.75.6 and BL.1 sub-variants showed a 69% confidence value for the S protein analysis, while it showed 78% for the complete genome. 

**Figure 7:**
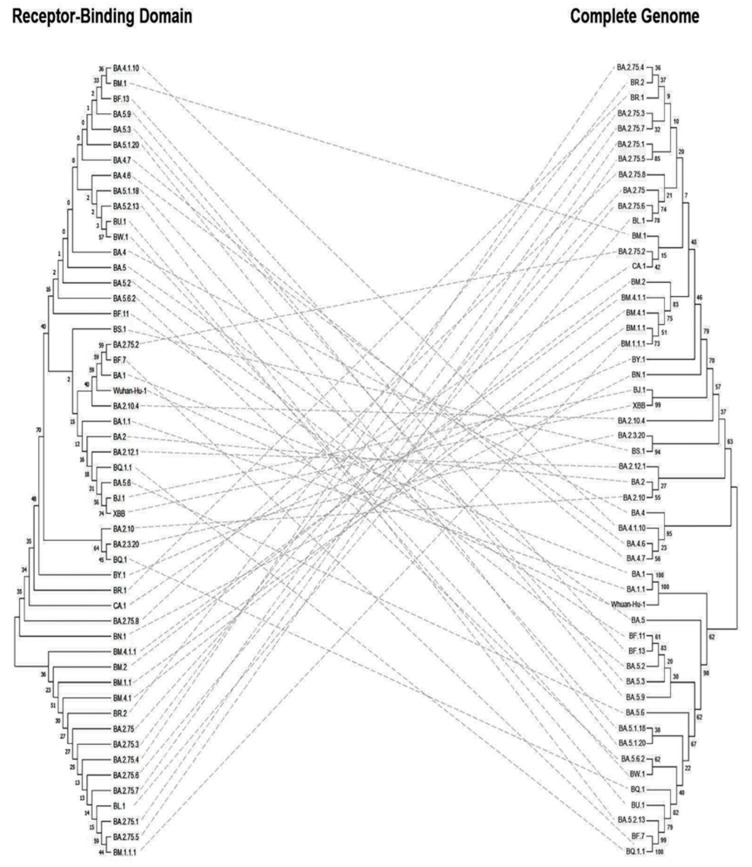
Phylogenetic trees for RBD (**left**) against the complete genome (**right**) of SARS-CoV-2 Omicron sub-variants. Sequences were obtained from the GISAID EpiCoV database as shown in Table S1.

Based on the RBD phylogenetic analyses (Figure 7 left), sub-variants BM.4.1.1, BM.2, BM.1.1, BM.4.1, BR.2, BA.2.75, BA.2.75.3, BA.2.75.4, BA.2.75.6, BA.2.75.7, BL.1, BA.2.75.1, BA.2.75.5, and BM.1.1.1 shared the same ancestor while the rest of the sub-variants evolved from a different ancestor. In addition, the BJ.1 and XBB sub-variants were originated from the same tip node with a confidence interval of 74%, however, both BA.1 and BA.1.1 sub-variants showed lower levels of clustering. In general, the confidence values of the RBD phylogenetic tree were lower compared with the trees based on the S protein and the complete genome.

## 4. Discussion

SARS-CoV2 has been characterized by the recurrent discovery of distinct variants over time and geography [21,27], which were subsequently named as variants of concern (VOCs) by the *WHO* and heralded the start of a new stage of the pandemic. These newly emerging variants, which have multiple mutations in the receptor-binding motif and a 25 amino acid patch at the tip of the S protein that mediates interaction with the human ACE2 receptor, are the result of the natural selection of SARS-CoV-2 during subsequent passage in the host [28,29,30,31]. These changes in the SARS-CoV-2 genome together provide fitness advantages, including improved transmissibility, infectivity, altered tropism, modified pathogenicity, and escape from host immune response produced by vaccination or prior infection [12]. The Omicron sub-variant B.1.1.529 was initially discovered in South Africa and Botswana around 23 months after the first case of COVID 19 was recorded. On 26 November 2021, the *WHO* designated the Omicron variant as a VOC [32,33,34].

Increasing evidence has shown that the Omicron variant has a significant transmissibility and a strong binding to the human ACE2 receptor [35,36,37], attenuated viral replication [38,39,40,41], and a reduced severity of illness in COVID-19 patients [42,43], as well as having a high level of environmental stability [44]. Notably, these mutations provide resistance to the majority of therapeutic antibodies [45,46,47,48], diminish the capacity of animal models to elicit an immunological response, and it may evade neutralising antibodies [49,50,51,52,53,54]. 

The rapid spread of the Omicron variant has been associated with an abrupt increase in the number of SARS-CoV-2 infections, catalysing the fourth wave of the pandemic in many countries, worldwide [33]. With the widespread effort to understand the impact of the SARS-CoV-2 Omicron variant, there is a need for the distillation of literature from original research sources into an accessible format for the community. A sudden rise in SARS-CoV-2 infections has been linked to the Omicron variant’s fast dissemination, sparking the fourth wave of the epidemic in several countries throughout the world [33]. Here, we summarize the most recent research on the SARS-CoV-2 omicron variant based on the scientific information that has been published to date and identify knowledge gaps that need to be filled up by additional research. As the pandemic progresses, we expect to offer a scientific support for monitoring and public health efforts to combat the SARS-CoV-2 Omicron sub-variants. Compared with earlier SARS-CoV-2 variants, the SARS-CoV-2 Omicron variant has a significant number of mutations in the S protein.

The number of cumulative substitutions in the analysed sites have shown that RBD is the most variable region with 163 mutations, whereas the C-terminal, especially the pre S1/S2 subunit, has 33 mutations and showed more conservation. Although there is no direct interaction between the NTD and the hACE2, the region showed high levels of variability as mutations in this region are involved in epitope recognition [55] and this region is the main target for antibodies [56]. The RBD and NTD have been identified as the S protein’s primary regions to produce an immune response [5]. Meanwhile, the regions that are not directly involved in binding with the hACE2 do not require any mutations to be more transmissible. The evolution process selects the variants that are able to complete the infection cycle quicker with a high replication rate [31,57].

The deduced amino acid analyses showed that the mutation G339D within BA.1, BA.1.1, BA.2, BF.7, and BQ.1.1 sub-variants that affects class 4 neutralising antibodies due to a change in the surface charge distribution from Gly, an uncharged non-polar amino acid, to Asp, a negatively charged amino acid [58,59]. This is favourable for the RBD-hACE2 binding [7,60]. The longer chain of Glu in G339E (BS.1) is preferred by proteins to bind with the hACE2 [61], meaning that it may bind stronger than G339D. In addition, mutations at site 346 may be able to reduce the effectiveness of neutralising antibodies [62]. The WT site interacts with ACE2 N450 via two hydrogen bonds and any substitution would result in a shorter and non-cationic sidechain, dissolving the interactions except for R346K at the BA.1.1 sub-variant [63]. Most of the Omicron sub-variants show a concerning evolution towards Ser (group 2, group 3, group 6, BQ.1, BR.2, and BM.2) and Thr (BA.2.75.1, BA.2.75.6, BF.7, BM.4.1.1, BQ.1.1, BA.2.75.2, and BL.1) [63].

Mutations including S371L (BA.1 and BA.1.1), S373P (BA.1, BA.1.1, BA.2 group 5, BA.2.75.1, BA.2.75.2, BA.2.12.1, BA.2.75.4, BA.2.75.6, BA.2.75.7, BL.1, BF.7, and BQ.1.1) and S375F (BA.1, BA.1.1, BA.2, group 5, BA.2.75.1, BA.2.75.2, BA.2.12.1, BA.2.75.4, BA.2.75.6, BA.2.75.7, BL.1, BF.7, and BQ.1.1) can cause conformational and flexibility changes in the S protein although they are outside the receptor-binding motif (RBM) [59]. The mutation sites were flexible when compared with the wild type (WT), causing an evasion of antibody binding to the hairpin loop [58]. A change of polarity (group 1) causes the formation of a cluster that causes biochemical changes in the RBD, allowing escape from class 4 antibodies and some from classes 1, 2, and 3 [8]. WT residue at 376 position represents Thr, which is a polar and neutral amino acid. Overall, the sub-variants showed mutations within non-polar and neutral residues (T376A, T376C, T376V, T376G, and T376F), however, two sub-variants (XBB and BJ.1) show mutation T376Y that maintains the original polarity.

The R408 residue forms a hydrogen bond with the glycan attached to N90 of hACE2 [64]. Therefore, a change in polarity or charge may alter the strength of the bond. In addition, the 417 site is located along the RBM and has a direct contact with the ACE2 [65] because it determines one of the most important interaction energies between the RBD and ACE2 [66]. The K417 reside within the Wuhan SARS-CoV-2 forms a salt bridge interaction with D30 of the ACE2 [58]. The change of amino acid from Lys-to-Asn (BA.1, BA.1.1, BA.2, group 1, BA.2.75.1, BA.2.12.1, BA.2.75.4, BA.2.75.6, BA.2.75.7, BL.1, BF.7, and BQ.1.1) causes the loss of interaction with the ACE2, due to a change of charge. On the other hand, this mutation causes loss of activity in class 1, 2, 3, and 4 antibodies, however, class 5 and 6 antibodies have been shown to be effective [67]. The 440-position found at a loop near the binding interface [65] causes a change of charge and has been shown to massively increase the interaction energy of the RBD with ACE2 [60], as well as an increased ability to escape neutralising antibodies [68]. It is also associated with an increased mortality [69]. Mutations including Asn-to-Lys (as seen in BA.1, BA.1.1, BA.2, group 5, XBB, BJ.1, BA.2.12.1, BA.2.75.4, BA.2.75.5, BA.2.75.7, BF.7, and BQ.1.1) cause a change of charge, leading to a positively charged interface. Previous studies reported that mutation of residue K444N (BU.1, BW.1, group 5, XBB, and BJ.1) was associated with increased mortality [69]. Mutation at residue G446S shows polarity changes, although it has been shown to decrease ACE2 binding [59]. Sub-variants that exhibit mutation at this residue (BA.1, BA.1.1, group 5, BA.2.75.1, BA.2.75.2, BA.2.75.4, BA.2.75.5, BA.2.75.7, and BL.1) demonstrate antibody escape. Residue 452 is located on the top of the RBD and is a target for neutralising antibodies [70]. Mutations of this residue to Met, Arg, or Gln have been shown to cause resistance to class 2 and 3 antibodies. Our results showed that mutation for this residue to Arg (group 1, BA.2.75.4, BF.7, and BQ.1.1) can increase the binding affinity to ACE2 by favoring adaptability and increasing the binding energy due to the replacement of a hydrophobic residue with a hydrophilic one. Therefore, it is considered one of the most detrimental mutations [71]. 

N460K/S residue (group 5, BA.2.75.1, BA.2.75.2, BA.2.10.4, BA.2.75.4, BA.2.75.5, BA.2.75.6, BA.2.75.7, and BQ.1.1) causes an escape of monoclonal antibodies, caused by the interface-destabilising effects of different binding energies [72]. The mutation of S477 residue from Ser-to-Asn (BA.1, BA.1.1, BA.2, group 5, BA.2.75.1, BA.2.75.2, XBB, BJ.1, BA.2.12.1, BA.2.75.4, BA.2.75.5, BA.2.75.6, BA.2.75.7, BL.1, BF.7, and BQ.1.1) creates hydrogen bonds with S19 and Q14 of ACE2 [58] that are associated with increased transmission and binding strength [59]. The 478 site is located within the interface and when mutated from Thr-to-Lys (BA.1, BA.1.1, BA.2, group 5, BA.2.75.1, BA.2.75.2, BA.2.12.1, BA.2.75.4, BA.2.75.5, BA.2.75.6, BA.2.75.7, BL.1, BF.7, and BQ.1.1) causes a change of surface charge, increasing the interaction with an extremely high binding energy and resisting neutralising antibodies [59,71]. The V483A mutation (BA.1, BA.1.1, BA.2, group 5, BA.2.75.1, BA.2.75.2, BQ.1, BA.2.12.1, BA.2.75.4, BA.2.75.5, BA.2.75.6, BA.2.75.7, BL.1, BF.7, and BQ.1.1) confers a stronger transmission capacity [18]. In addition, the mutation from Val- to-Phe (group 3 and group 6) causes a change of polarity, affecting the glycosylation of N343 or sugar positioning [73]. Residue E484 is located on the interface, and the Glu residue has not been found to interact directly with ACE2, but it is involved in the disruption of hydrogen bonds and salt bridge interactions with antibodies [5]. The change of surface charge distribution has a direct effect on antibody binding and interaction between RBD and ACE2 [65]. The E484A substitution (as observed in BA.1, BA.1.1, BA.2, group 5, BA.2.75.1, BA.2.75.2, BA.2.75.5, BA.2.75.6, BA.2.75.7, BL.1, BF.7, and BQ.1.1) contributes to affecting antibody neutralisation through a change in charge. This mutation also contributes to the increase in interaction [60,71]. The E484Q mutation (BA.2.10.4) increases the affinity for ACE2 and causes immune escape [5].

Another position (486) in the S protein forms a direct interact with the ACE2 [65] and its mutation from F486V (BF.7 and BQ.1.1) has been shown to impair the activity of class 1 and 2 antibodies [70]. The F486P substitution (group 1) is responsible for increasing the ACE2 binding affinity without losing immune resistance, causing a higher transmissibility [74]. The Q493 substitution is located at the top of the RBD (group 5, BA.2.75.1, BA.2.75.2, BA.2.75.4, BA.2.75.5, BA.2.75.6, BA.2.75.7, BL.1, BF.7, and BQ.1.1) and it interacts with ACE2-K31 and mediates the resistance to class 1 and 2 antibodies [70]. A mutation in residue at position Q493 to Arg (BA.1, BA.1.1, BA.2, group 1, BA.2.75.4, and BA.2.12.1) disrupts the interaction and creates a salt bridge with ACE2-R35, conferring additional strength [58,59]. Interestingly, the mutation to Arg is the most conserved change at this residue and it is mainly associated with Omicron variant mortality [69]. In addition, G496 can form a direct contact with ACE2 [65] and its mutation G496S (BA.1 and BA.1.1) leading to a formation of hydrogen bonds with ACE2-D38 due to the change in polarity and an increase in the binding [68]. 

For the Wuhan prototype strain of SARS-CoV-2, the Q498 residue forms Van der Waal interactions with ACE2-Y41, however, mutation from Gln-to-Arg (as we noticed in the BA.1, BA.1.1, BA.2, group 5, BA.2.75.1, BA.2.75.2, BA.2.12.1, BA.2.75.4, BA.2.75.5, BA.2.75.6, BA.2.75.7, BL.1, BF.7, and BQ.1.1) forms a hydrogen bond with ACE2-D38, which may increase the binding strength [56]. The position 501 located at the RBM within the interaction interface [65] is one of the key amino acid residues that determine the RBD-ACE2 interaction strength [71]. The N501 residue in Wuhan SARS-CoV-2 forms a Van der Waal interaction with ACE2-Q42 [58], however, the N501Y mutation (as observed in BA.1, BA.1.1, BA.2, group 5, BA.2.75.1, BA.2.75.2, BA.2.12.1, BA.2.75.4, BA.2.75.5, BA.2.75.6, BA.2.75.7, BL.1, BF.7, and BQ.1.1) forms a new stacking ring with ACE2-Y41 that strengthens the binding energy with a nine-fold increased affinity with the ACE2 [5]. The Y505 residue at Wuhan SARS-CoV-2 forms hydrogen bonds with ACE2-E37 and ACE2-R393. The mutation from Tyr-to-His (BA.1, BA.1.1, BA.2, group 5, BA.2.75.1, BA.2.75.2, BA.2.12.1, BA.2.75.4, BA.2.75.5, BA.2.75.6, BA.2.75.7, BL.1, BF.7, and BQ.1.1) results in a positive surface charge, disrupting the hydrogen bonds [58] and decreasing the ACE2 binding. The NTD interface contains about 40 residues, of which nine are analysed in the current study (T19, G142, V143, Y144, Y145, H146, K147, W152, and G257). These sites play a key role in NTD binding with antibodies. Therefore, a mutation has the potential to cause immune escape [75]. The most conserved mutation among all sub-variants is T19I that reduces the affinity of S protein binding [76]. The T19I, together with L24S and G142D, are responsible for the evasion of neutralising antibodies [77,78]. None of the analysed sub-variants in this study had shown all these three mutations simultaneously. However, they display T19I with either L24S or G142.

On the other hand, the furin cleavage site is important for SARS-CoV-2 pathogenicity, membrane fusion, and replication [79] and mutation in this site leads to immune escape and increased transmissibility [80]. Previous reports have shown that T547K mutation (BA.1 and BA.1.1) increases the S1-S2 interaction, creating a more compact protein [81]. Omicron sub-variants with the D614G mutation (BA.1, BA.1.1, and group 3) leads to enhanced host-to-host transmission and infectivity [82]. The N658 residue strongly interacts with furin protease, promoting cell cleavage, fusion, and entry [83]. Pro-to-His mutation at site 681 has been shown to increase the cleavability at the S1/S2 junction, which has a direct impact on the viral entry, while infectivity is not well known but can mediate the cell fusion [84,85]. In addition, this mutation (Pro-to-His) could cause a conformational change that influences the nearby residues indirectly [86]. According to the in vitro study conducted by Kuzmina et al. [87], mutation Pro-to-Arg (BJ.1, XBB and BS.1) can enhance the cell fusion and production of syncytia, induced by the S protein, which is typically seen in COVID-19 patients who have developed severe chronic respiratory disease [88]. The P681S mutation (group 2) was initially detected only in one Sub-variant (B.1.1), however it is one of the most conserved residues in this site. The P681S mutation represents a possible immune escape [89]. The amino acid analyses for the S2 subunit revealed that mutations N764K and N856K provide cleavage sites for SKI-1/S1P and prevent the internal fusion peptide from being exposed to the membrane fusion and syncytia formation. The protein convertase SKI-1/S1P is expressed in the bronchus and nasopharynx but not in the lungs, which may explain differing tissue tropisms [90,91,92,93,94]. Sub-variants BA.1 and BA.1.1 showed N764K and N856K mutations, whereas BA.2, group 3, and group 7 showed only N764K.

The phylogenetic analyses revealed high confidence levels of clustering between groups of sub-variants, such as BA.1 and BA.1.1 or XBB and BJ.1. It was interesting to investigate how these sub-variants share the same mutation patterns. The evolution and substitution analyses suggest that the Omicron sub-variants demonstrate high clustering levels and may have the same immune escape and binding properties. As seen in the cryo-EM structure, most mutations in the key residues are located in the binding interface with the hACE2. Omicron has higher levels of transmission, compared with previous variants, as a result of biological evolution and mutations in the key residues. All of the analysed sub-variants showed at least one mutation that had avoided the neutralisation of monoclonal antibodies. Therefore, these immune-evading sub-variants gained an immunity advantage via antigenic drift, meaning that they are able to disrupt the bonds with antibodies produced post-infection or through vaccination.

The development of novel Adoptive Cell Transfer (ACT) approaches led researchers and clinicians to highly efficient technologies based on genetically engineered T lymphocytes, with chimeric antigen receptor (CAR)-T cells which is a modern trend for the treatment of viral infections, including SARS-CoV-2 [95]. Moreover, frequent changes in the SARS-CoV-2 genome and the subsequent structural reshaping of key epitopes are strongly associated with the restricted development of universal SARS-CoV-2 vaccines. Heterologous prime-boost vaccination regimens, the construction of chimeric immunogens, the design of protein nano-particle antigens, and the use of conserved neutralising epitopes are four innovations from several domains that broaden neutralising antibody protection against variants. The heterologous prime-boost regimen is a ready-to-use yet passive or temporal hysteresis method that responds to variances among all the methods discussed above. That is to say, variant-specific vaccinations will only be created when new variations appear [96]. 

By combining several immunogens or conserved neutralising epitopes into a single particle, chimeric antigen constructs and antigens delivered by nanoparticles demonstrate superiority in fending against developing variations. Purification of recombinant protein subunits is the key approach employed in these two procedures, enabling large-scale manufacturing while also highlighting the significance of creating an advantageous protein expression system. The neutralising epitopes identified in S2 exhibit great exploration value to serve as targets for universal vaccines, which has been proven to be conserved among coronaviruses, or at least within SARS-CoV-2 variants, despite being less sensitive to inducing effective neutralising antibodies than RBD or S [96].

It is recommended to incorporate novel elements into universal vaccine design that can give broader immune responses, such as vaccinations that generate powerful cellular responses [97,98]. Protein subunit vaccines created using conserved T-cell epitopes from the S, N, and M proteins have a great potential for use as universal vaccinations [99,100]. Moreover, immunisations administered intramuscularly are less likely to prevent viral transmission at the upper respiratory tracts [101]. A nasal vaccination that replicates the natural infection process can help vaccines by inducing a mucosal immune response (mostly sIgA) in respiratory tracts [102,103].

## 5. Conclusions

This study provides valuable insights into the binding of the S protein of the Omicron sub-variants with hACE2. It offers a first head-to-head comparison of the Omicron sub-variants and thus helps tracking the spread and mutations along the evolutionary trajectories of SAR-CoV-2. The finding highlights critical areas of mutations across the major Omicron sub-variants (over 50) which can lead to antibody escape, increased affinity to hACE2, and in some cases, a correlation with increased mortality. In conclusion, this study highlights the importance of continuous evolution, and proposes several hotspots in the S proteins of SARS-CoV-2 sub-variants to train the future design and development of COVID-19 vaccines.

## Data Availability

All data is provided either in the main or in Supplementary Material.

## Supplementary Materials

The following supporting information can be downloaded at: https://www.mdpi.com/article/10.3390/vaccines11030668/s1, Figure S1: Amino acid mutations (blue) of the residues of interest in the S gene of each Omicron variant compared with the Wuhan-Hu-1 variant. S protein regions are labelled at the top; Table S1. Virus name, accession ID, and country of Omicron sub-variants studied in this study. The data was obtained from GISAID’s EpiCoV database; Figure S2: Localisation of amino acid RBD substitutions in cryo-electron microscopy 3D structure model of the Wuhan-Hu-1′s S protein with RBD in the prefusion conformation, top view (accession ID: 6SVB). (A) Most conserved residues; (B) most variable residues. Data adapted from Figure 3. Not all conserved residues are labelled due to missing residues in the amino acid sequence; Table S2. Grouping of Omicron sub-variants based on the mutations, key features, and percentage of mutations in the N-terminal domain; Figure S3: Localisation of amino acid RBD substitutions in the cryo-electron microscopy 3D structure model of the Wuhan-Hu-1′s S protein with RBD in the prefusion conformation, top view (accession ID: 6SVB) of sub-variants BA.1, BA.1.1, BA.2, BA.2.10, BA.2.10.4, BA.2.12.1., BA.2.3.20, BA.2.75, BA.2.75.1. The structure was produced via PyMoL, the mutations are designated with a contrasting colour and labelled; Table S3. Grouping of Omicron sub-variants based on the mutations, key features, and percentage of mutations in the RBD; Figure S4: Localisation of amino acid RBD substitutions in the cryo-electron microscopy 3D structure model of the Wuhan-Hu-1′s S protein with RBD in the prefusion conformation, top view (accession ID: 6SVB) of sub-variants BA.2.75.2, BA.2.75.3, BA.2.75.4, BA.2.75.5, BA.2.75.6, BA.2.75.7, BA.2.75.8, BA.4, BA.4.1.10. The structure was produced via PyMoL, the mutations are designated with a contrasting colour and labelled; Table S4. Grouping of Omicron sub-variants based on the mutations, key features, and percentage of mutations in the pre S1/S2 subunit; Figure S5: Localisation of amino acid RBD substitutions in the cryo-electron microscopy 3D structure model of the Wuhan-Hu-1′s S protein with RBD in the prefusion conformation, top view (accession ID: 6SVB) of sub-variants BA.4.6, BA.4.7, BA.5, BA.5.1.18, BA.5.1.20, BA.5.2, BA.5.2.13, BA.5.3, BA.5.6. The structure was produced via PyMoL, the mutations are designated with a contrasting colour and labelled; Table S5. Grouping of Omicron sub-variants based on the mutations, key features, and percentage of mutations in the S2 subunit; Figure S6: Localisation of amino acid RBD substitutions in the cryo-electron microscopy 3D structure model of the Wuhan-Hu-1′s S protein with RBD in the prefusion conformation, top view (accession ID: 6SVB) of sub-variants BA.5.6.2, BA.5.9, BF.11, BF.13, BF.7, BJ.1, BL.1, BM.1, BM.1.1. The structure was produced via PyMoL, the mutations are designated with a contrasting colour and labelled. Figure S6: Localisation of amino acid RBD substitutions in the cryo-electron microscopy 3D structure model of the Wuhan-Hu-1′s S protein with RBD in the prefusion conformation, top view (accession ID: 6SVB) of sub-variants BM.1.1.1, BM.2, BM.4.1, BM.4.1.1, BN.1, BQ.1, BQ.1.1, BR.1, BS.1. The structure was produced via PyMoL, the mutations are designated with a contrasting colour and labelled. Figure S7: Localisation of amino acid RBD substitutions in the cryo-electron microscopy 3D structure model of the Wuhan-Hu-1′s S protein with RBD in the prefusion conformation, top view (accession ID: 6SVB) of sub-variants BR.1, BR.2, BW.1, BY.1, CA.1, XBB. The structure was produced via PyMoL, the mutations are designated with a contrasting colour and labelled.
