# Supplementary material for: Deep Structural Analysis of Myriads of Omicron Sub-Variants Revealed Hotspot for Vaccine Escape Immunity
Source: Vaccines (Basel). 2023 Mar 15;11(3):668. doi: 10.3390/vaccines11030668 (PMC10059128; doi:10.3390/vaccines11030668)
Supplement: Supplementary file 1 [file vaccines-11-00668-s001.zip › vaccines-2267638-SM/Table S3.pdf]

**Table S3.** Grouping of Omicron sub-variants based on the mutations, key features, and percentageof mutations in the RBD.

| Group Number | Sub-variant(s) | Key Features | Percentage of Mutations Carried |
|---|---|---|---|
| 1 | BA.4, BA.4.1.10, BA.4.6, BA.4.7, BA.5, BA.5.1.18, BA.5.1.20, BA.5.2, BA.5.2.13, BA.5.3, BA.5.6, BA.5.6.2, BA.5.9, BF.11, BF.13, BM.1 | Mutations shared withother variants. | 100% |
| 2 | BM.1.1, BM.1.1.1, BM.4.1,BY.1, CA.1 | Similar to group 3; distinctive mutationV483S. | 89.3% |
| - | BM.2 | Similar to group 2 and 3; distinctive mutation V483X. | 89.3% |
| 3 | BA.2.75.8, BA.2.3.20, BR.1 | Similar to group 2; distinctive mutation V483F. | 89.3% |
| - | BR.2 | Similar to group 2 and 3; distinctive mutationV483I. | 89.3% |
| 4 | BU.1, BW.1 | Shows mostly unique mutations when compared to other variants. | 96.4% |
| 5 | BA.2.75, BA.2.75.3 | Similar to BA.75.1 and BA.75.2; distinctive mutation N440K. | 57.1% |
| - | BA.2.75.1 | Similar to group 5 and BA.275.2; distinctive mutation K417N. | 57.1% |

| | | | |
|---|---|---|---|
| - | BA.2.75.2 | Similar to group 5 and BA.2.75.1. | 57.1% |
| 6 | BN.1, BA.2.10 | Similar to BQ.1; distinctive mutation V483F. | 89.3% |
| - | BQ.1 | Similar to group 6; distinctive site V483. | 85.7% |
| 7 | XBB, BJ.1 | Shows mostly unique mutations when comparedto other variants. | 100% |
| - | BA.2.10.4 | Shows many unique mutations when compared to other variants. | 85.7% |
| - | BA.2.12.1 | Similar to variants BA.2.75.4 and BA.2.75.7; distinctive sites G446, L452Q, N460, F486 and Q493R. | 57.1% |
| - | BA.2.75.4 | Similar to variants BA.2.12.1 and BA.2.75.7; distinctive sites G446S, L452R, N460K, F486 and Q493. | 60.7% |
| - | BA.2.75.5 | Mutations shared with other variants. | 42.9% |
| - | BA.2.75.6 | Similar to BL.1; distinctive sites G446 and N460K. | 53.6% |

| | | | |
|---|---|---|---|
| - | BA.2.75.7 | Similar to variants BA.2.75.4 and BA.2.12.1; distinctive sites G446S, L452, N460K, F486S and Q493. | 60.7% |
| - | BF.7 | Similar to BQ.1.1; distinctive mutationsK444T and N460K. | 71.4% |
