# Supplementary material for: Deep Structural Analysis of Myriads of Omicron Sub-Variants Revealed Hotspot for Vaccine Escape Immunity
Source: Vaccines (Basel). 2023 Mar 15;11(3):668. doi: 10.3390/vaccines11030668 (PMC10059128; doi:10.3390/vaccines11030668)
Supplement: Supplementary file 1 [file vaccines-11-00668-s001.zip › vaccines-2267638-SM/Table S4.pdf]

Table 4. Grouping of Omicron sub-variants based on the mutations, key features, and percentageof mutations in the pre S1/S2 subunit.

Group Number	Sub-variant(s)	Key Features	Percentage of Mutations Carried
1	BA.2.10, BA.2.3.20, BA.2.75.8, BM.1.1, BM.1.1.1, BM.2, BM.4.1, BN.1, BQ.1, BR.1, BR.2, BY.1, CA.1	Shows mostly unique mutations when comparedto other variants.	100%
2	BA.4, BA.4.1.10, BA.4.6, BA.4.7, BA.5, BA.5.1.18, BA.5.1.20, BA.5.2, BA.5.2.13, BA.5.3, BA.5.6, BA.5.6.2, BA.5.9, BF.11, BF.13, BM.1	Shows mostly unique mutations when comparedto other variants.	100%
3	BA.2.12.1, BA.2.75, BA.2.75.1, BA.2.75.2, BA.2.75.3, BA.2.75.4, BA.2.75.5, BA.2.75.6, BA.2.75.7, BF.7, BL.1, BM.4.1.1, BQ.1.1	Similar to BA.2; distinctive mutation H655Y. Shows mostly unique mutations when compared to other variants.	66.7%
-	BA.2	Similar to group 3; distinctive mutation H655T.	50%
4	BJ.1, XBB	Shows mostly unique mutations when comparedto other variants.	100%
5	BU.1, BW.1	Shows mostly unique mutations when compared to other variants.	83.3%
-	BS.1	Shows mostly unique mutations when comparedto other variants.	100%

-	BA.2.10.4	Shows mostly unique mutations when compared to other variants.	100%
-	BA.1	Similar to BA.1.1; distinctive mutation H655Y.	83.3%
-	BA.1.1	Similar to BA.1.; distinctive mutation H655T.	83.3%
