# Supplementary material for: Deep Structural Analysis of Myriads of Omicron Sub-Variants Revealed Hotspot for Vaccine Escape Immunity
Source: Vaccines (Basel). 2023 Mar 15;11(3):668. doi: 10.3390/vaccines11030668 (PMC10059128; doi:10.3390/vaccines11030668)
Supplement: Supplementary file 1 [file vaccines-11-00668-s001.zip › vaccines-2267638-SM/Table S5.pdf]

Table S5. Grouping of Omicron sub-variants based on the mutations, key features, and percentageof mutations in the S2 subunit.

Group Number	Sub-variant(s)	Key Features	Percentage of Mutations Carried
1	BA.4.1.10, BA.4.6, BA.4.7, BA.5, BA.5.1.18, BA.5.1.20, BA.5.2, BA.5.2.13, BA.5.3, BA.5.6, BA.5.6.2, BA.5.9, BF.11, BF.13, BM.1	Similar to BA.4; distinctive mutationN764G.	100%
-	BA.4	Similar to group 1; Distinctive mutation N764L.	100%

2	BM.1.1, BM.1.1.1, BM.2, BM.4.1, BN.1, BQ.1, BR.1, BR.2, BY.1, CA.1	Similar to group 7; distinctive mutation N764L.	77.8%
3	BA.2.75.2, BA.2.75.3, BA.2.75.4, BA.2.75.5, BA.2.75.6, BM.4.1.1, BQ.1.1, BF.7	Similar to group 4 and variants BA.2.12.1 and BA.2.75; distinctive mutation N764K.	44.4%
4	BA.2.75.1, BA.2.75.7, BL.1	Similar to group 3 and variants BA.2.12.1 and BA.2.75; distinctive siteshowing no mutation N764.	33.3%
-	BA.2.75	Similar to group 3 and 4and variant BA.2.12.1; distinctive mutation N764L.	44.4%
-	BA.2.12.1	Similar to group 3 and 4and variant BA.2.75; distinctive mutation N764E.	44.4%
5	BJ.1, XBB	All unique mutations except D796F, sharedwith group 6.	100%
6	BU.1, BW.1	Shows many unique mutations when compared to other variants.	88.9%

7	BA.2.10, BA.2.3.20, BA.2.75.8	Similar to group 2; distinctive mutationN764K.	77.8%
8	BA.1, BA.1.1	Unique mutations N856Kand L981F. The rest of the mutations are shared with other variants.	66.7%

-	BA.2	Mutations shared with other variants.	33.3%
-	BS.1	Shows many unique mutations when compared to other variants.	88.9%
-	BA.2.10.4	Shows many unique mutations when comparedto other variants.	88.9%
