# Supplementary material for: Deep Structural Analysis of Myriads of Omicron Sub-Variants Revealed Hotspot for Vaccine Escape Immunity
Source: Vaccines (Basel). 2023 Mar 15;11(3):668. doi: 10.3390/vaccines11030668 (PMC10059128; doi:10.3390/vaccines11030668)
Supplement: Supplementary file 1 [file vaccines-11-00668-s001.zip › vaccines-2267638-SM/Table S1.pdf]

**Table S1.** Virus name, accession ID and country of Omicron sub-variants studied in this study. The data was obtained from GISAID's EpiCoV database.

| Variant | Virus Name | Accession ID | Country of Sequence |
|---|---|---|---|
| Wuhan-Hu-1 | hCoV-19/Wuhan/Hu-1/2019 | EPI_ISL_402125 | China |
| BA.1 | hCoV-19/Russia/MOW-CRIE-L188V0599/2021 | EPI_ISL_11584122 | Russia |
| BA.1.1 | hCoV-19/Finland/THL-202209082/2022 | EPI_ISL_11813207 | Finland |
| BA.2 | hCoV-19/India/MH-INSACOG-CSIR-NEERI1403/2022 | EPI_ISL_11883942 | India |
| BA.2.10 | hCoV-19/USA/VA_FBCH_3454/2022 | EPI_ISL_15748451 | USA |
| BA.2.10.4 | hCoV-19/England/LSPA-15209982/2022 | EPI_ISL_15019850 | England |
| BA.2.12.1 | hCoV-19/India/Karnataka/KA-INSACOG_JNC-294/2022 | EPI_ISL_13409765 | India |
| BA.2.3.20 | hCoV-19/Singapore/11876/2022 | EPI_ISL_15031207 | Singapore |
| BA.2.75 | hCoV-19/Nepal/NPHL-S-1845/2022 | EPI_ISL_15313041 | Nepal |
| BA.2.75.1 | hCoV-19/India/HR-764002196/2022 | EPI_ISL_14819052 | India |
| BA.2.75.2 | hCoV-19/India/HR-764774549/2022 | EPI_ISL_14841683 | India |
| BA.2.75.3 | hCoV-19/India/KA-INSACOG_JNC_319/2022 | EPI_ISL_14722979 | India |
| BA.2.75.4 | hCoV-19/India/KA-INSACOG_JNC_320/2022 | EPI_ISL_14722980 | India |
| BA.2.75.5 | hCoV-19/India/JK-759101892/2022 | EPI_ISL_15192541 | India |
| BA.2.75.6 | hCoV-19/Nepal/NPHL-S-1829/2022 | EPI_ISL_15313025 | Nepal |
| BA.2.75.7 | hCoV-19/India/KA-INSACOG_JNC_343/2022 | EPI_ISL_14723003 | India |
| BA.2.75.8 | hCoV-19/New Zealand/22XA3062/2022 | EPI_ISL_15278276 | New Zealand |

| | | | |
|---|---|---|---|
| BA.4 | hCoV-19/USA/TN-SPHL-3729/2022 | EPI_ISL_15852538 | USA |
| BA.4.1.10 | hCoV-19/Spain/PV-HUD-78252035/2022 | EPI_ISL_15311814 | Spain |
| BA.4.6 | hCoV-19/France/HDF-IPP47842/2022 | EPI_ISL_14439420 | France |
| BA.4.7 | hCoV-19/France/HDF-IPP47913/2022 | EPI_ISL_14439355 | France |
| BA.5 | hCoV-19/France/HDF-IPP47946/2022 | EPI_ISL_14439279 | France |
| BA.5.1.18 | hCoV-19/New Zealand/22YA3038/2022 | EPI_ISL_15276713 | New Zealand |
| BA.5.1.20 | hCoV-19/Canada/BC-BCCDC-514682/2022 | EPI_ISL_15184271 | Canada |
| BA.5.2 | hCoV-19/France/OCC-IPP48063/2022 | EPI_ISL_14439359 | France |
| BA.5.2.13 | hCoV-19/Denmark/DCGC-588970/2022 | EPI_ISL_15267531 | Denmark |
| BA.5.3 | hCoV-19/Netherlands/NH-RIVM- 103407/2022 | EPI_ISL_13760344 | The Netherlands |
| BA.5.6 | hCoV-19/USA/OH-CDC-QDX41381270/2022 | EPI_ISL_15113965 | USA |
| BA.5.6.2 | hCoV-19/Canada/BC-BCCDC-506289/2022 | EPI_ISL_15051942 | Canada |
| BA.5.9 | hCoV-19/Netherlands/NH-RIVM- 104967/2022 | EPI_ISL_13855261 | The Netherlands |
| BF.11 | hCoV-19/USA/NY-URMC-220989081-1/2022 | EPI_ISL_15145141 | USA |
| BF.13 | hCoV-19/USA/NJ-CDC-LC0886409/2022 | EPI_ISL_15160036 | USA |
| BF.7 | hCoV-19/Brazil/ES-LACENES-321304402/2022 | EPI_ISL_15604583 | Brazil |
| BJ.1 | hCoV-19/Canada/BC-BCCDC-500338/2022 | EPI_ISL_15050448 | Canada |
| BL.1 | hCoV-19/Nepal/NPHL-S-1887/2022 | EPI_ISL_15430092 | Nepal |

| | | | |
|---|---|---|---|
| BM.1 | hCoV-19/France/IDF-HMN-22102060308/2022 | EPI_ISL_15554243 | France |
| BM.1.1 | hCoV-19/USA/NY-NYULH9255/2022 | EPI_ISL_15710478 | USA |
| BM.1.1.1 | hCoV-19/Bangladesh/icddrb-G148987/2022 | EPI_ISL_15157642 | Bangladesh |
| BM.2 | hCoV-19/USA/CA-SCPHL-22WGS02101/2022 | EPI_ISL_15533693 | USA |
| BM.4.1 | hCoV-19/Hong Kong/HK-HKPU- PUU046841104/2022 | EPI_ISL_15680668 | Hong Kong |
| BM.4.1.1 | hCoV-19/India/GA-ICMR-NIV- INSACOG-GSEQ-12561/2022 | EPI_ISL_15854770 | India |
| BN.1 | hCoV-19/Malaysia/MKAKK_WC414/2022 | EPI_ISL_15757370 | Malaysia |
| BQ.1 | hCoV-19/USA/CA-Stanford-103_S46/2022 | EPI_ISL_15078219 | USA |
| BQ.1.1 | hCoV-19/Brazil/ES-LACENES-321312063/2022 | EPI_ISL_15814053 | Brazil |
| BR.1 | hCoV-19/USA/SC-CDC-LC0899124/2022 | EPI_ISL_15411882 | USA |
| BR.2 | hCoV-19/Japan/IC-14890/2022 | EPI_ISL_15472231 | Japan |
| BS.1 | hCoV-19/Japan/TY41-768-P1/2022 | EPI_ISL_15492872 | Japan |
| BU.1 | hCoV-19/USA/CT-CDC-LC0903675/2022 | EPI_ISL_15410608 | USA |
| BW.1 | hCoV-19/Netherlands/NH-AUMC- 034050/2022 | EPI_ISL_15826475 | The Netherlands |
| BY.1 | hCoV-19/India/TN-CDFD-O-111/2022 | EPI_ISL_15469908 | India |
| CA.1 | hCoV-19/USA/NC-CDC-LC0894816/2022 | EPI_ISL_15399812 | USA |
| XBB | hCoV-19/USA/NJ-CDC-LC0923611/2022 | EPI_ISL_15744367 | USA |
