# Supplementary material for: Deep Structural Analysis of Myriads of Omicron Sub-Variants Revealed Hotspot for Vaccine Escape Immunity
Source: Vaccines (Basel). 2023 Mar 15;11(3):668. doi: 10.3390/vaccines11030668 (PMC10059128; doi:10.3390/vaccines11030668)
Supplement: Supplementary file 1 [file vaccines-11-00668-s001.zip › vaccines-2267638-SM/Table S2.pdf]

Table S2. Grouping of Omicron sub-variants based on the mutations, key features, and percentageof mutations in the N-terminal domain.

Group Number	Sub-variant(s)	Key Features	Percentage of Mutation sCarried
1	BQ.1.1, BA.2, BA.2.12.1	Similar to groups 2 and 3;distinctive sites show no mutations: K147, W152, F157, I210.	11.5%
2	BA.2.75.1, BA.2.75.5	Similar to groups 1 and 3; distinctive sites are G142,and G257X.	26.9%
3	BL.1, BA.2.75.3, BA.2.75.4, BA.2.75.6, BA.2.75.7	Similar to groups 1 and 2.	30.8%
4	BM.1, BA.4, BA.4.1.10, BA.4.6, BA.4.7, BA.5, BA.5.1.20, BA.5.2, BA.5.2.13, BA.5.3, BA.5.6, BA.5.6.2, BA.5.9, BF.11, BF.13	Distinctive mutation fromBA.5.1.18 is T19I.	100%
-	BA.5.1.18	Similar to group 4; distinctive mutationT19X.	100%
5	BU.1, BW.1	Shares residues 19-143 and 213 with groups 6, 7 and 8.	100%
6	BM.1.1, BM.4.1, BR.2	Similar to groups 7 and 8; distinctive mutations T19Iand Y144E.	96.2%
-	BA.2.75.8	Similar to group 6; distinctive mutation T19X.	96.2%

7	BR.1, CA.1, BM.2, BN.1	Similar to groups 6 and 8; distinctive mutation Y144X.	96.2%
-	BM.1.1.1	Similar to group 7; distinctive mutation Y144G.	96.2%
8	BQ.1, BA.2.3.20, BA.2.10	Similar to groups 6 and 7; distinctive mutation Y144K.	96.2%
9	BJ.1, XBB	Share the same mutations in positions 19-95.	92.3%
-	BS.1		100%
-	BA.2.10.4		100%
-	BA.1	Only two mutations A67V, and T95I.	7.7%
-	BA.1.1	Unique mutations H69L, and L212I.	30.7%
-	BA.2.75	Unique mutations H69L, V70I, G142X, V143X, and L212I.	34.6%
-	BA.2.75.2	Mutations shared with other variants.	15.4%
-	BF.7	Mutations shared with other variants.	7.7%

-	BM.4.1.1	Mutations shared with other variants.	26.9%
-	BY.1	Mutations shared with other variants.	92.3%
