# Supplementary figures and images for: Deep Structural Analysis of Myriads of Omicron Sub-Variants Revealed Hotspot for Vaccine Escape Immunity
Source: Vaccines (Basel). 2023 Mar 15;11(3):668. doi: 10.3390/vaccines11030668 (PMC10059128; doi:10.3390/vaccines11030668)

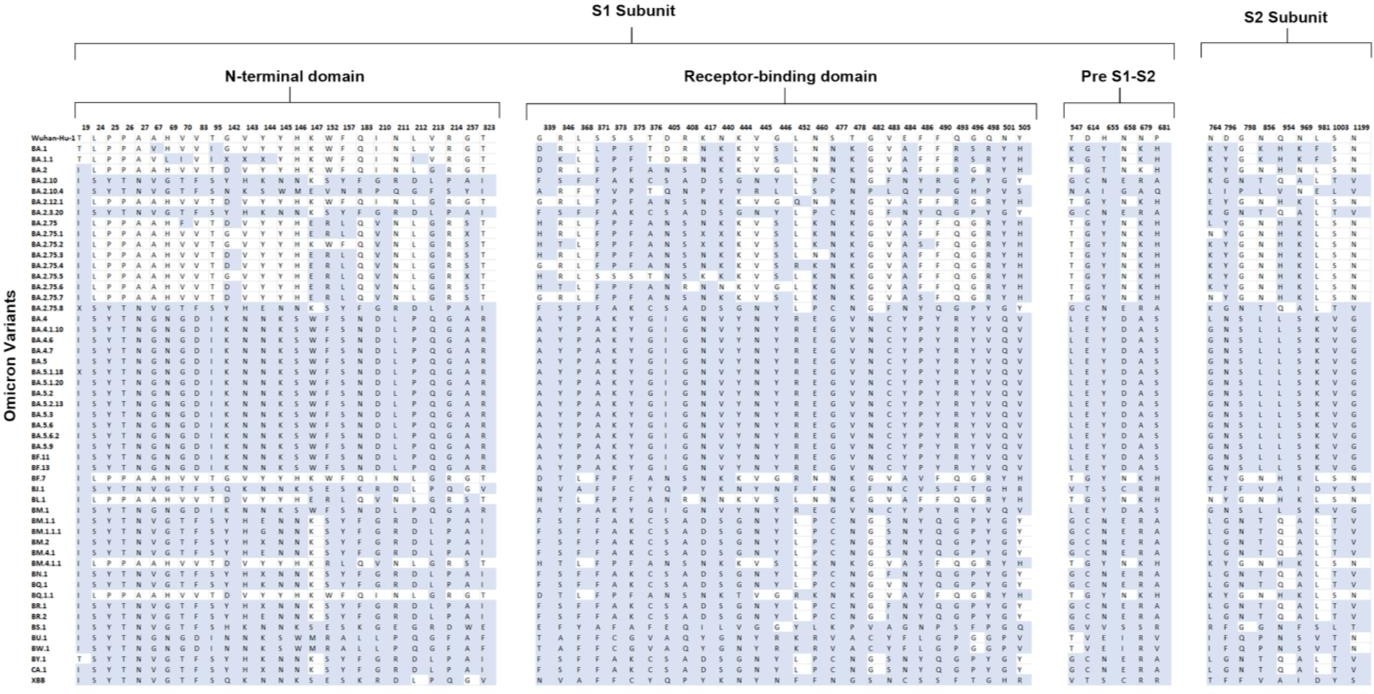

Supplement: Supplementary file 1 [file vaccines-11-00668-s001.zip › vaccines-2267638-SM/Figure S1.jpg]

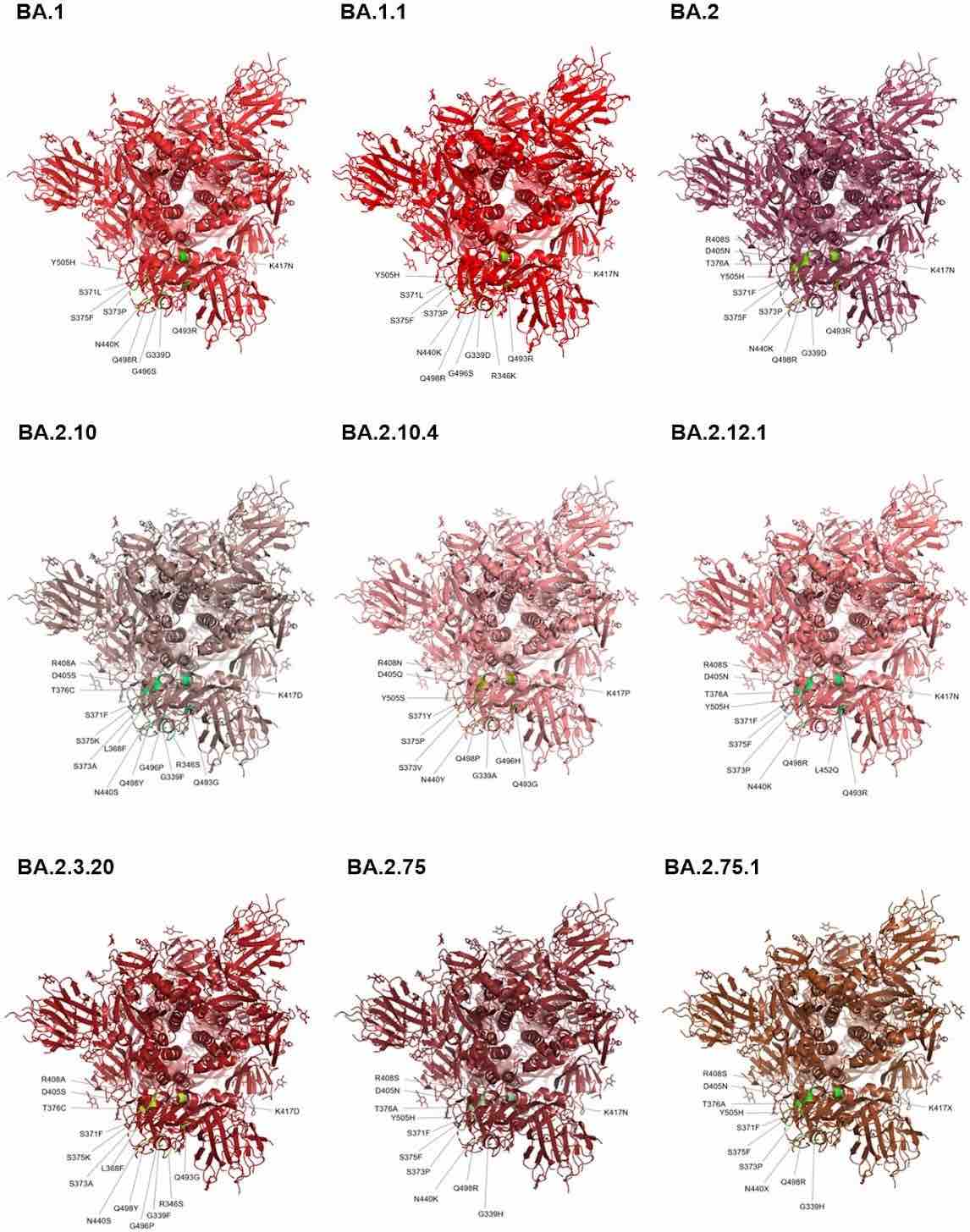

Supplement: Supplementary file 1 [file vaccines-11-00668-s001.zip › vaccines-2267638-SM/Figure S2.jpg]

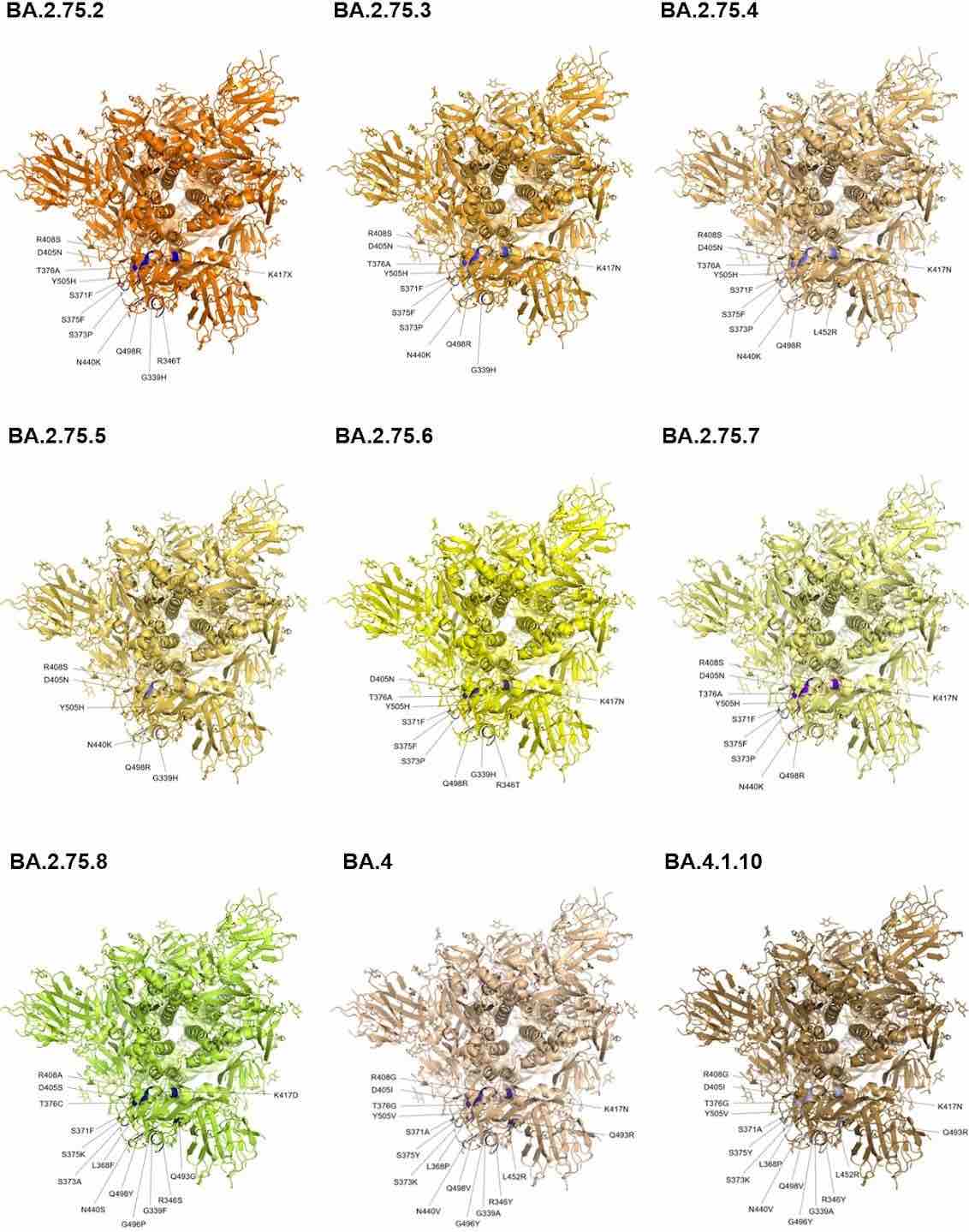

Supplement: Supplementary file 1 [file vaccines-11-00668-s001.zip › vaccines-2267638-SM/Figure S3.jpg]

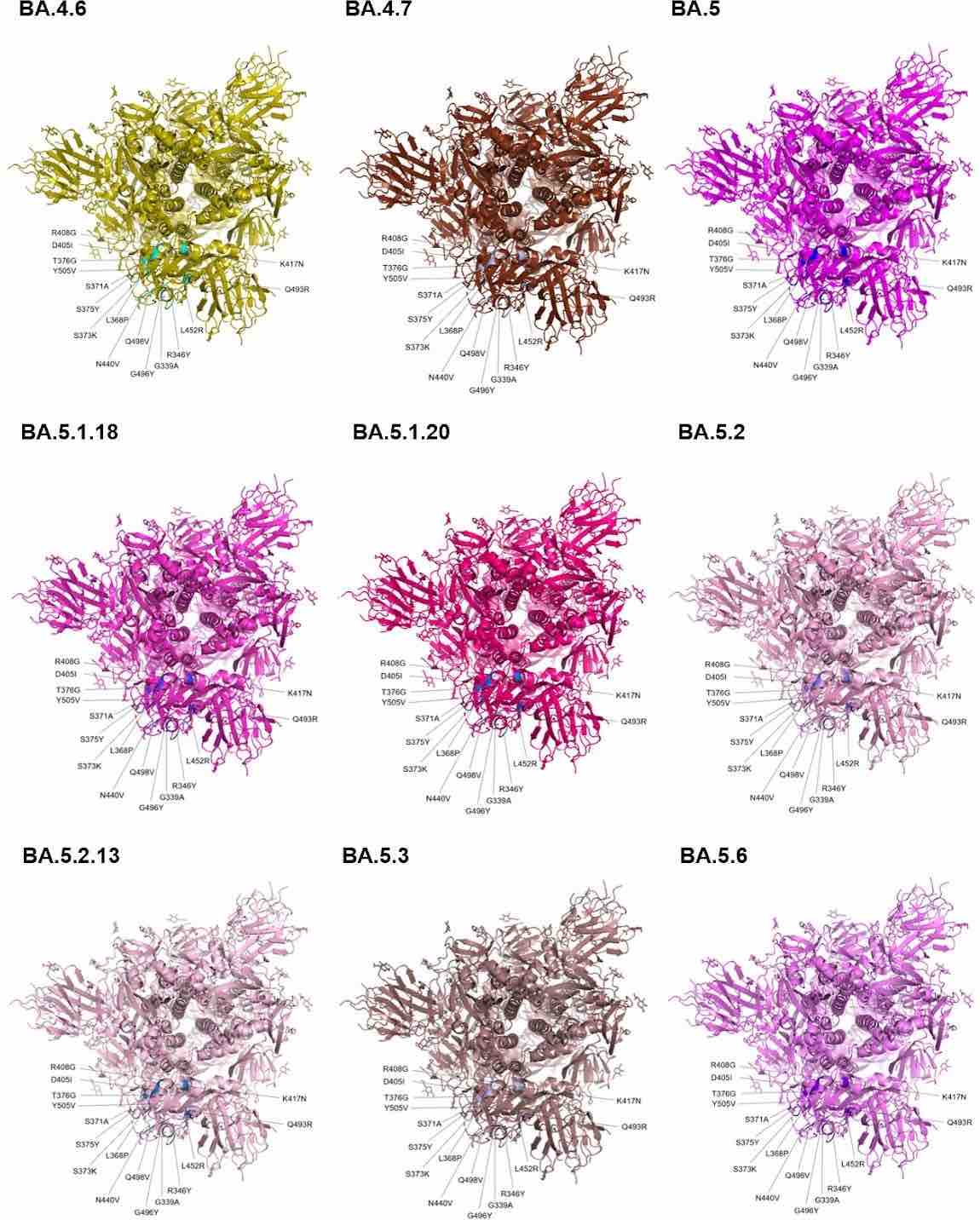

Supplement: Supplementary file 1 [file vaccines-11-00668-s001.zip › vaccines-2267638-SM/Figure S4.jpg]

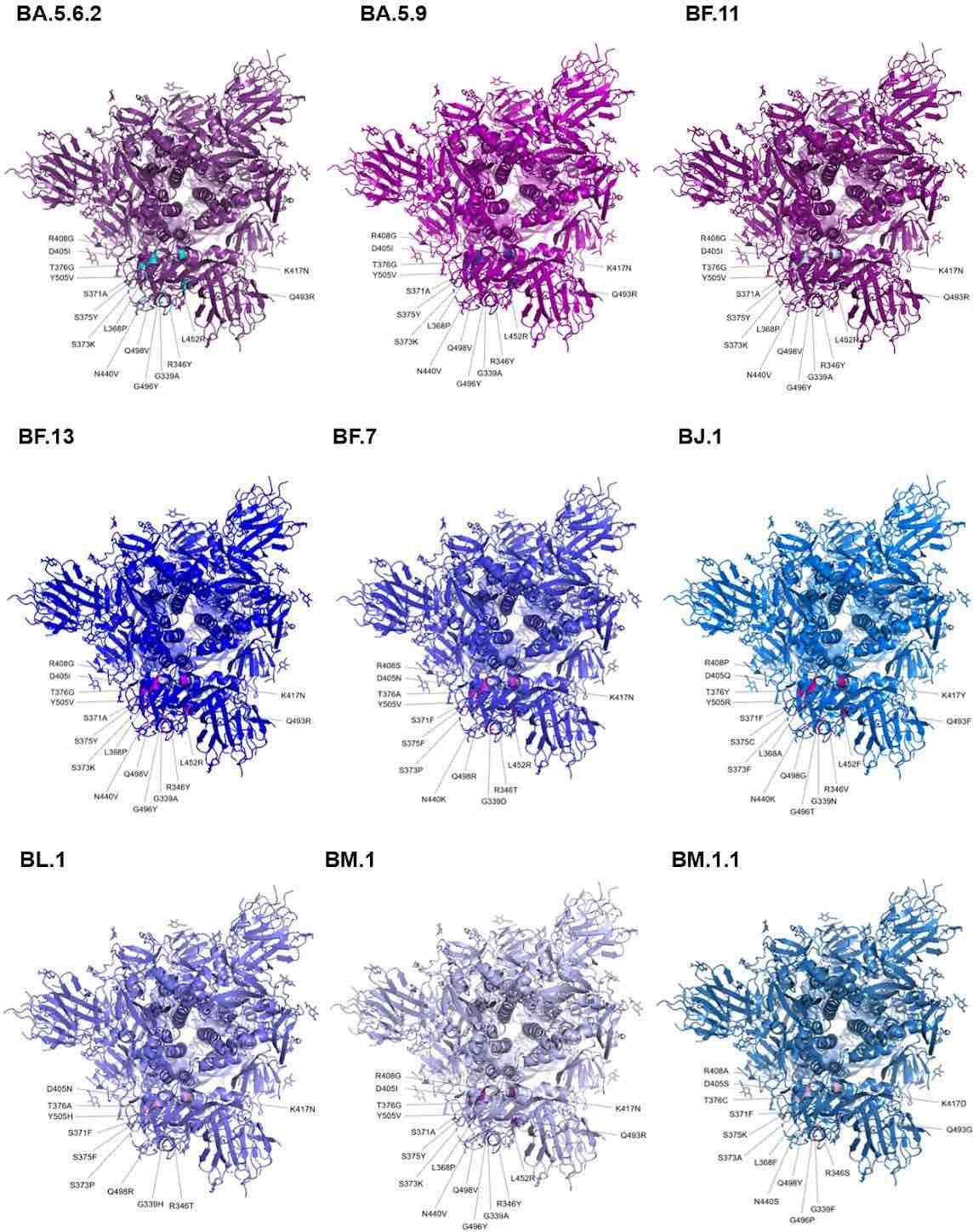

Supplement: Supplementary file 1 [file vaccines-11-00668-s001.zip › vaccines-2267638-SM/Figure S5.jpg]

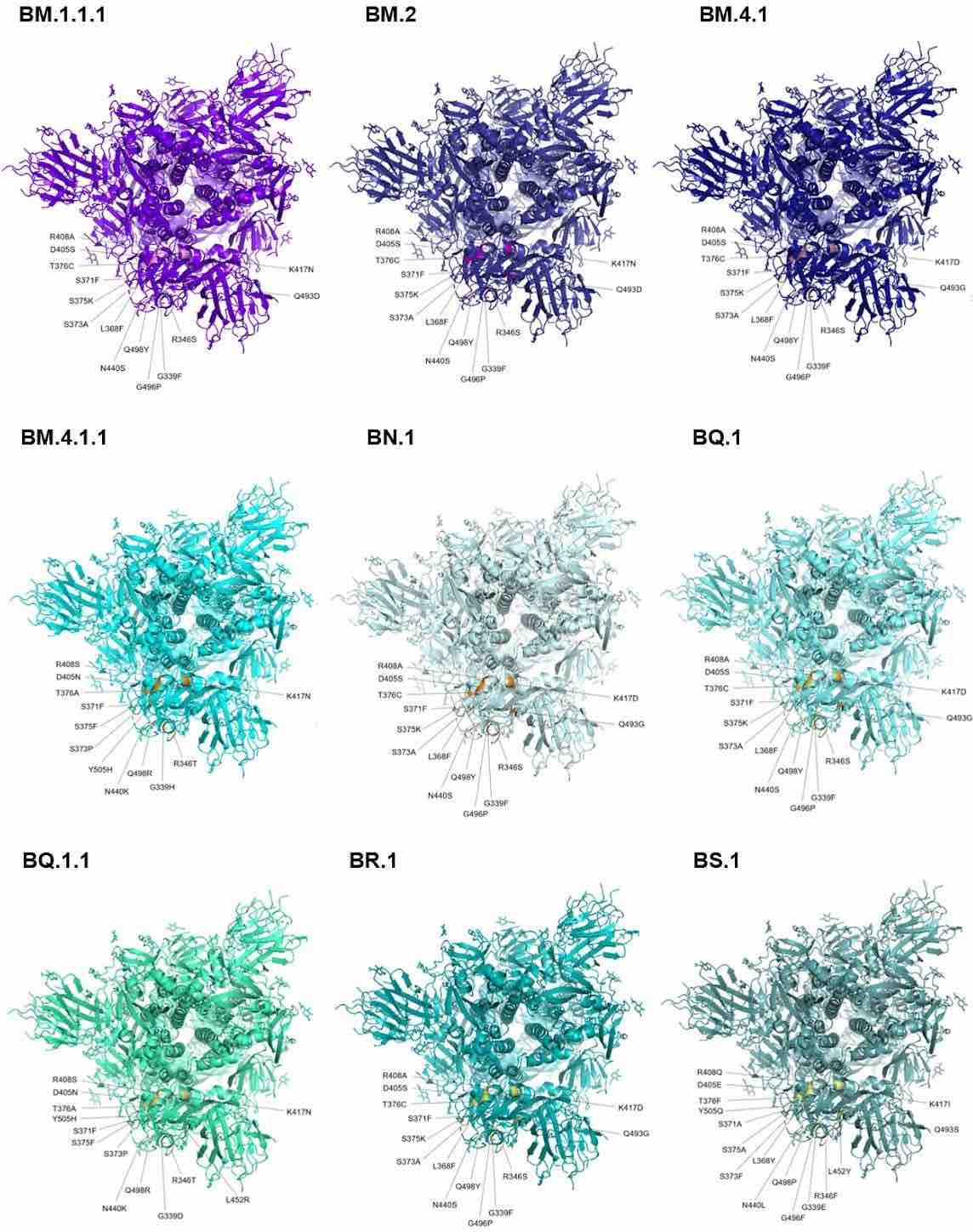

Supplement: Supplementary file 1 [file vaccines-11-00668-s001.zip › vaccines-2267638-SM/Figure S6.jpg]

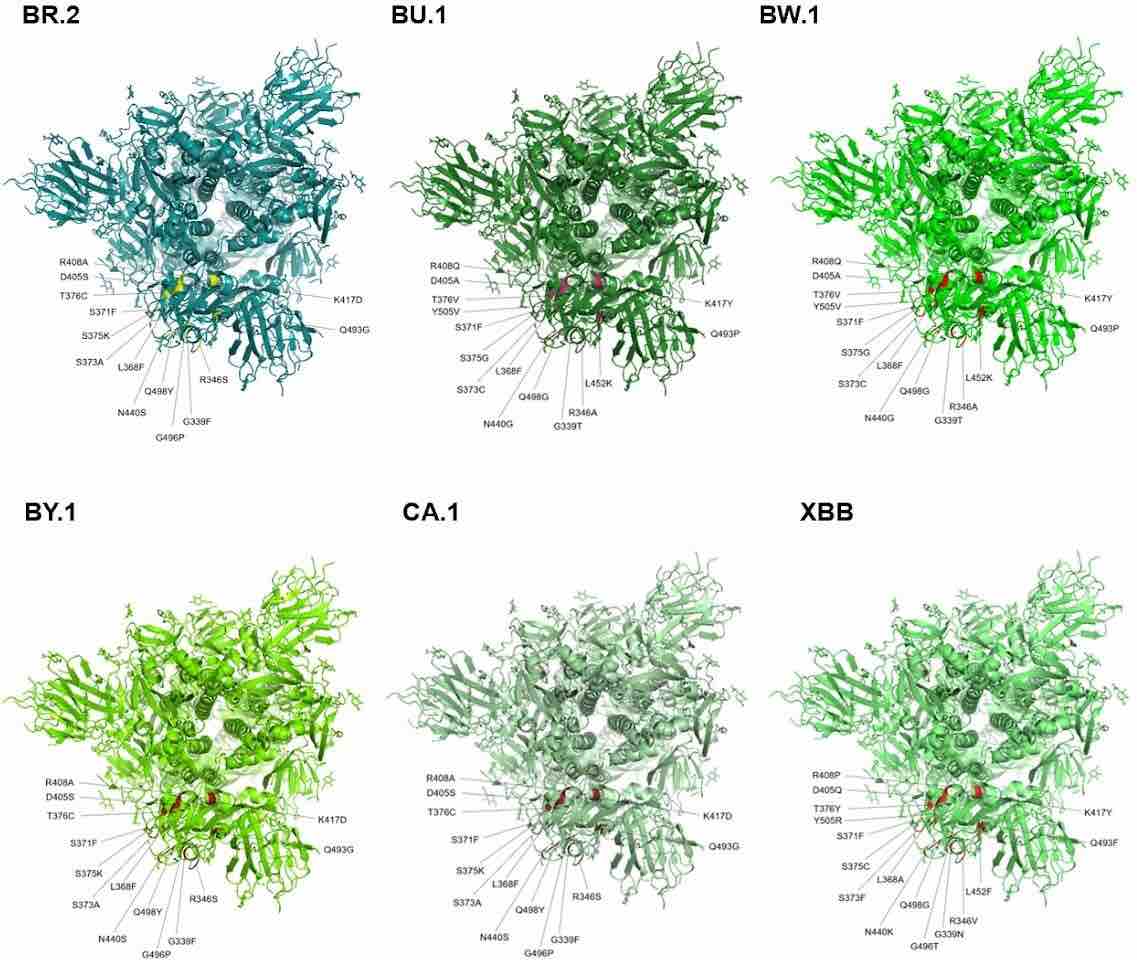

Supplement: Supplementary file 1 [file vaccines-11-00668-s001.zip › vaccines-2267638-SM/Figure S7.jpg]
